# Elizabeth Casson Memorial Lecture 2024: The time is now, building a social movement to demonstrate the value of occupation

**DOI:** 10.1177/03080226251370180

**Published:** 2025-10-29

**Authors:** Katrina Bannigan

**Affiliations:** Research Assistant, Scottish Learning Disabilities Observatory, School of Health and Wellbeing, University of Glasgow, Glasgow, UK

**Keywords:** Healthy ageing, health promotion, occupational therapy, primary prevention, public health, social movement

## Abstract

There is a pervading feeling of dissonance within the occupational therapy profession: a sense that occupational therapy, and the work of occupational therapists, is not always recognised. Alongside this, there are examples of occupational therapists whose work is recognised at the highest levels and who are realising Elizabeth Casson’s legacy. The social age, which we now live in, provides the means to capitalise on examples such as these, to change this narrative, through social movements and social leadership. By committing to a social movement, a form of collective action that enables occupational therapists to tell their own stories through their own networks, and exercising social leadership, which is not dependent on hierarchy or position, occupational therapists can promote the centrality of occupation in the lives of people. Collective action is contingent on individuals making a commitment to act, even if this requires overcoming any barriers they may experience. Elizabeth Casson’s contribution to the profession and the collective wisdom of the Elizabeth Casson lectures provide inspiration for anyone unsure of where to begin.

## The time is now

I deliberately named this lecture ‘The time is now’ rather than ‘Our time is now’. Davis (2010: 14) wrote an article, in, called ‘Our time is now’. In it, she argued, ‘Our time has come – our time is now’ saying ‘There has never been a more exciting time for occupational therapy practitioners than right now! As this new decade begins, the foundation of our profession, occupation-based practice, is perfectly aligned with the world of health care, both nationally and internationally’ . However, there is danger in thinking that our time is now. It suggests that we have one moment and, if we miss it, our time has passed. The centrality of occupation in the lives of people is a truth; it is not a time dependent concept, and it is true for all time. By committing to a social movement and exercising social leadership – by doing whatever you can, in whatever way you can to share the story of occupational therapy – you are not one voice but part of a crescendo of voices that will ensure the centrality of occupation in the lives of people is a given. Once others recognise this truth, we can stop being absorbed with how we are perceived as a profession and can direct our energies towards the people and communities we serve. The time is now; we all need to act now. The time is now for the world to recognise that we are enacting one of the greatest ideas of the 20th century for the benefit of people and communities. We should not be ‘waiting passively for the go-getters to lead the way’ (Tempest and Dancza, 2019: 602); we need to emulate Elizabeth Casson and take advantage of the opportunities provided by the Social Age we live in. If we want things to change, not acting is not an option.

### Occupation’s value

Reilly (1962: 5), in her Eleanor Clarke Slagle lecture, asserted that ‘occupational therapy can be one of the great ideas of 20th-Century medicine’. Events, in the time since, have underscored the truth of her assertion. The International Classification of Functioning, Disability and Health, the World Health Organisation’s (2002) framework for disability and health, positioned participation – involvement in a life situation – as essential to health. Occupation is the building block of participation because our involvement in life situations is rooted in our engagement in activities that are important to us – our meaningful occupations – and, in turn, our occupations are rooted in our hopes, goals and aspirations for the future. This resonates with the message from The Ottawa Charter for Health Promotion (WHO, 1986: para. 3) which affirmed ‘To reach a state of complete physical, mental and social well-being, an individual or group must be able to identify and to realize aspirations, to satisfy needs, and to change or cope with the environment’. For the global population, the COVID-19 pandemic demonstrated the importance of our everyday occupations to well-being and living well. We also, now, understand occupation’s value beyond medicine and its role in social transformation (Schiller et al., 2023); ‘as a vital force to meet society’s needs’ (Schwartz, 2009: 681). After all, it is ‘Through occupation, people can develop and maintain their families, neighbourhoods and communities as sources of belonging, opportunities and common action’ (Laliberte Rudman et al., 2019: 316).

### Dissonance within the profession

**Figure 1.**
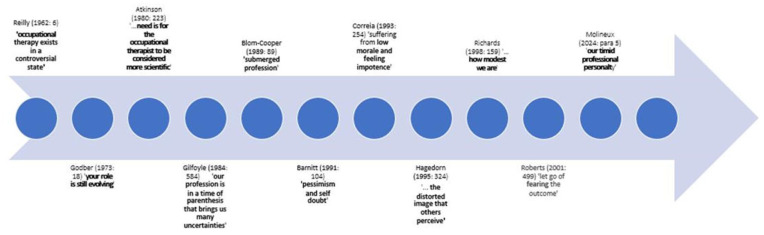
Examples of concerns about the profession that have been expressed over the years.

Developments such as these means, in many ways, it has never been a more exciting time to be an occupational therapist. Yet, there is a pervading feeling of dissonance within the profession. As recently as 2021, RCOT’s chief executive, Steve Ford, observed that the profession feels like ‘the underdog’ with other professions being better at grasping opportunities (Samuels, 2021). Whilst this feels qualitatively different to the latent anxiety described by Reilly (1962: 5), ‘about our value as a service to sick people’, it reflects a continuing concern within the profession that has been expressed in various ways over the years (Figure 1). It speaks to Pollard’s (2018: 489) contention that ‘Occupational therapists have still to seize their heroic destiny and realise the power within their central concept’. Yet, at the same time, there is also a heightened sense within the profession of the value of occupational therapy; we have a much greater understanding of how our work, focussing on meaningful occupation, transforms lives and communities (Schiller et al., 2023). This can be seen in promotional campaigns, such as RCOT’s Occupational Therapy Week campaign to ‘Own your superpower’ (Samuels, 2023), or WFOT’s work with the World Health Organisation about the contribution occupational therapists can make to humanitarian efforts in response to crises and disasters (WFOT, 2024). When occupational therapists make forays into new areas of practice, such as primary care, improvements are observed in activity levels, independence, health and lifestyle skills as well as cost savings (RCOT, 2024). Nonetheless, the feeling remains that occupational therapy, and the work of occupational therapists, is not always recognised (Samuels, 2023). This is important because, if there was greater recognition of the centrality of occupation, more people and communities could benefit from occupational therapy (Mickel, 2019). In essence, our ‘power as a profession comes from being recognised for what we do’ (Padilla, 2017: xiii).

The current struggle to have our expertise – our contribution – be recognised appears to be rooted in a lack of confidence (Samuels, 2023), rather than the anxiety that Reilly (1962) described. My experience of working with people at all levels of the profession supports this observation. For example, recently I have been asked by students at a Leadership Showcase ‘What advice would you give to individuals lacking the confidence to take on a leadership role?’. An RCOT Special Interest Group invited me to present a keynote about ‘How to overcome a lack of confidence and not putting ourselves forward’. This could sound gloomy if it was not for the fact that there are incredible occupational therapists who are being successful and whose work is recognised. For example, until January of this year, every Chief Allied Health Professions Officer in the United Kingdom was an occupational therapist. Dr Musharrat Ahmed-Landeryou was nominated for a National Diversity Award, Dee Christie, Chair of the Elizabeth Casson Trust, was awarded an OBE for services to occupational therapy and Dr Niina Kolehmainen has just been commissioned to conduct a £2.3 million trial to assess how parents and professionals can support young children with neurodisability to develop independence in everyday self-care activities. Occupational therapists were award winners in five of the nine AHP awards – including Allied Health Professional of the Year – last year, namely

- The Creative provision of placements award was awarded to Professor Lisa Taylor for Peer Enhanced e-Placements.
- The Public health champion award was awarded to the occupational therapy team at Sport for Confidence.
- The research impact award was awarded to Dr Natalie Jones for her research work related to occupational therapy home visits and breakfast clubs for stroke patients, and
- The Greener AHP award was awarded to Sue Norman for the implementation of the electric urgent response van, which promotes healthy aging for older people living with frailty by reducing falls, long lies and avoidable admissions.
- Occupational therapists working in practice, like Karen Robertson, are securing fellowships, and Joshua Ige has had a programme of service improvement in occupational therapy and mental health published in the *BMJ Open Quality* (Ige and Hunt, 2022).

Clearly not all occupational therapists feel like ‘underdogs’. These are all examples of how individual occupational therapists in their everyday work are ensuring that occupational therapy is being recognised for the contribution it can make.

Before we move on to the central theme of this lecture, I want to take a moment to consider what I hope you will take away from it. First, I want to position myself, as the person who is giving this lecture, to provide some context. I am a white female occupational therapist who has predominantly worked in academia in the global north and I acknowledge I am speaking from a place of privilege as a white, working class, non-disabled, heterosexual and cisgender woman. I also need to be honest in that I will not be saying anything new, although I will endeavour to say it in a way that inspires you to commit to realising Elizabeth Casson’s legacy. We need to stop focussing on concerns about how the profession is perceived so we can concentrate our efforts on the work we need to do. I invite you to stop thinking about this lecture as an entity, or how I am shaping up as an Elizabeth Casson Memorial Lecturer. I want you to think about the ideas I am sharing and what they mean for your work as an occupational therapist. What commitment are you going to make to being part of a social movement? How are you going to ensure that centrality of occupation in people’s lives is universally recognised? What I ask of you, I ask of myself, and I will share how I plan to be part of this social movement by increasing the recognition of occupational therapy within primary prevention, using an example of occupation-based practice in public health.

## A social movement for a Social Age?

The focus of this lecture is how we use the opportunities provided by the Social Age we live in to capitalise on individual successes of occupational therapists, such as those I have described, to increase the recognition of the centrality of occupation. The Social Age is an age characterised by ‘change: changes in how we work, how we learn, how we lead, how we connect and communicate’ (Stodd, 2014a: para. 1). It is an age in which work is not constrained by desks and buildings, the boundaries between work and home are blurred, 9 to 5 Monday to Friday working is no longer typical and where it is as easy to connect with someone in another country as it is with our neighbour (Stodd, 2014a). If we are no longer constrained by hierarchies or organisations, engaging with communities and developing networks becomes more important (Stodd, 2014b). In the Social Age, networks are community based, their communication is mediated through technology; they are more agile, capitalise on diverse skills, involve relationships centred around stories and can be highly responsive to events (Stodd, 2014b). It has often been asserted if we want change it is up to us, as occupational therapists, to achieve it (e.g., Hammond 2014; Hunter, 2013; Preston, 2022) and living in the Social Age really does mean shaping the perception of occupational therapy is in our hands (Hinojosa, 2007). One way we can promote the centrality of occupation in the lives of people is by committing to a social movement.

Social movements – which cover all kinds of issues for example the Civil Rights Movement in the United States or the anti-war movement – are a form of collective action where ‘collective action consists of any goal-directed activity engaged in jointly by two or more individuals’ (Snow et al., 2018: 5). They use the power of networks motivated by a common direction (a shared story) to shape and change society (Stodd, 2014b). They have been formally defined as

‘Collectivises acting with some degree of organization and continuity outside of institutional or organizational channels for the purpose of challenging or defending extant authority, whether it is institutionally or culturally based, in the group, organization, society, culture, or world order of which they are a part’. (Snow et al., 2018: 10)

By engaging the collective resources of the profession, capitalising on the power of networking, we can create a social movement to ensure that the centrality of occupation in the lives of people and communities is recognised.

### Sharing stories about our work

We can tell our own stories, share stories about our work with the people and communities we work with and have influence over what services are commissioned or funded. We do not have to be constrained by, or rely upon, the hierarchies or organisations, we work in, to recognise, or tell, our story. Tempest and Dancza (2019: 603) have already discussed ‘social leadership’ in the Social Age, as a way for occupational therapy to influence and have ‘our voices heard within and beyond organisational hierarchies’. Social leadership makes it easier for everyone, at whatever stage of their careers, whatever their fears, to embrace leadership and influence change. Social movements and social leadership also resonate with Pollard’s (2018: 487) observation ‘that occupational therapy is a global network with the population of a city, and thus represents a community that can be a vibrant voice’.

## Elizabeth Casson: A hero of her time?

This feels like a good point to turn our attention to Elizabeth Casson, the truly remarkable woman in whose honour these lectures are named. Previous Elizabeth Casson Memorial Lecturers have reflected on her significant contribution to the profession and, while I am not sure what I can add, I can recommend Mountain’s (2005) erudite exposition, especially for those new to Elizabeth Casson’s story. Mountain (2005) demonstrated how we can draw on Elizabeth Casson’s legacy as a source of strength for grappling with the challenges we face today. I agree with Nick Pollard’s (2018: 488) assessment that ‘Elizabeth Casson is an occupational therapy hero’. But one reason why I am reluctant to rehearse her story is that we need to be careful not to hero worship her, or others who have gone before, such as Eleanor Clarke Slagle, Sylvia Docker, Muriel Driver, Margaret Rutherford, or Vona du Toit. This is not to diminish their memory or achievements; the profession, occupational therapy and the value of occupation would not be what it is today without them. However, there is a danger that by overly focussing on their achievements, we venerate them and believe we cannot emulate them. This may explain why Tempest and Dancza (2019) warned us against becoming passive, waiting for the go-getters to take the lead. If we are to make a lasting ‘breakthrough’, and move from feeling like the underdog to being recognised, we all need to act. We all have a part to play, no matter how small or large, in shaping the narrative about occupation and occupational therapy.

### A social movement inspired by Elizabeth Casson?

Elizabeth Casson was a woman of her time. Her world was smaller; the places she visited and was inspired by, Gartnavel Hospital in Scotland, Bloomingdale Hospital in New York and Boston School of Occupational Therapy (Wilcock, 2004), covered most of the occupational therapy world then. Today, as well as the wider geographical spread of the profession, we also can no longer expect one person – one voice – to successfully span the many aspects of professional life: policy, management and administration, practice, education and research. The agenda is just too big. The Elizabeth Cassons that live amongst us today are achieving great things, but usually in one aspect of professional life. It means we need to move away from a reliance on individuals, or our professional bodies (Hinojosa, 2007), to promote the value of occupation. ‘Collective action is more powerful than individual action in supporting a social movement’ (Lawson-Porter, 2009: 292). We have benefited enormously in the past from the energy and actions of individual people but in this bigger, changed world we need collective action, which requires us to operate in different ways. Just as Elizabeth Casson harnessed the tools of her age we need to do the same. Everyone has to play their part. Social movements – driven by stories, using social media to communicate through networks, formed of fluid and adaptive groups, that change as they need to, and not limited by geography – are one way to increase our impact.

### The need for collective action

As a person born before the internet existed, I am the last person to suggest what our social movement to promote the value of occupation should look like. There are multiple possibilities that can emerge from diverse voices, different groups, networks of people and artificial intelligence – but how these all come together or interact, to form a social movement, is up to us collectively to imagine and reimagine. RCOT’s current work around communities and connections, although not framed in the context of a social movement, speaks to this (Fuentes Tibbitt et al., 2023). We have great stories to tell. Can you imagine how many more people would know about occupational therapy if each and every occupational therapist working in the United Kingdom today had all posted on social media about each of the occupational therapists I described above? It would have generated more than half a million posts; the reach of a global city (Pollard, 2018). Even if each of us had just posted about the Allied Health Professional of the Year (NHS England, 2024) that would have been 40,000 opportunities to share a story about an occupational therapist. Did you know that the Allied Health Professional of the Year is an occupational therapist? And what it is that she has achieved? If not, perhaps after what you learn in this lecture you could post about how proud you are of Sue Norman’s achievement? Share the story of how her greener solution to reducing falls and promoting healthy ageing exemplifies occupational therapy. If you also tag in your communities, we will have formed a community network focused on the achievement of an occupational therapist. This network may not come together again in the same way until the next national achievement of an occupational therapist. This is how social movements start and operate. Tagging in the people and communities you work with, or want to influence, not just other occupational therapists, is essential. If we don’t do this, it means we are just talking amongst ourselves. If we are just talking amongst ourselves, then it is unsurprising that the value of occupation is not recognised.

## What will you do?

If collective action is required for us to have reach and influence, it means we all have a part to play in creating a social movement. Making the choice to act, or not, matters. For everyone who chooses not to share the story of the centrality of occupation, it lessens the impact of our profession. Our strength is in our numbers; that’s the power of the collective voice. I appreciate in choosing not to act, people will explain their choice in terms of the barriers they experience.

### We need to have courage

**Figure 2.**
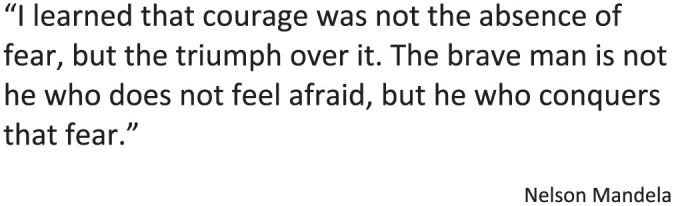
A lack of confidence is not the issue.

A lack of confidence is understandable because we all experience a lack of confidence, at least some of the time. However, a lack of confidence is not the issue (Figure 2). The issue is letting a lack of confidence shape what we do, or how we respond to events. We need to have courage. If you lack confidence, remember ‘we become brave by doing brave acts’ (Brown and Gillespie, 1997: 108). As Drummond (2010: 295) observed, when discussing research capacity in her Casson Memorial lecture, ‘You don’t know until you try’. As occupational therapists, we expect the people and communities we work with to have the courage to push beyond their comfort zones. Our acts do not need to be grandiose; a series of small acts with a range of communities makes a difference when we are all acting collectively. For example, posting on social media or sharing the stories of successful occupational therapists with your colleagues, communities and networks, to help them understand the work occupational therapists do, will increase the reach and impact of our work (Jacobs, 2012).

### Knowing where to start

**Figure 3.**
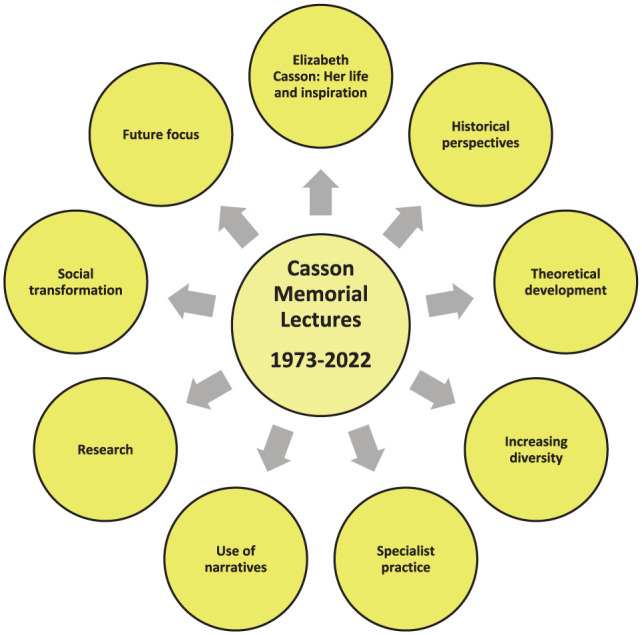
A summary of the ideas encapsulated in the Casson Memorial lectures.

Another concern I have heard expressed is ‘Not knowing where to start’. If you are not sure where to start, the Casson lectures, and others such as the Eleanor Clarke Slagle lectures, are a rich resource of ideas and provide an excellent starting point. In preparing for this lecture, I have read every Casson lecture and, I can honestly say whilst they are not all captivating, I am stunned by what an incredible resource they are for the profession. They provide a powerful insight into the profession in the United Kingdom. As well a strong sense of Elizabeth Casson’s life and inspiration, they include an historical perspective, theoretical developments, the importance of increasing diversity in the profession, descriptions of highly specialist practice, the value of narratives and a focus on social transformation and a strong future focus (see Figure 3). If a module was created, focussed solely on them, it would provide students with a strong grounding for working as an occupational therapist. It would cultivate their sense of the historical development of the profession, the importance of reading for learning, highlight the value of different writing styles to discuss the work of occupational therapists and provide them with an insight into the wide scope of practice and the range of career paths available to them. I feel a little disappointed in myself that it has taken this opportunity to motivate me to read them all sequentially. It has been a humbling and thought-provoking experience that has left me feeling very encouraged about occupational therapy in the United Kingdom. The Casson Memorial lectures are full of ideas – a treasure trove of inspiration – for how we can meet our hopes and aspirations as a profession. In reviewing the lectures, I have culled examples of ideas of where to start. Table 1 contains examples from each published lecture. You can see that the ideas span measurement, writing, building a community of influence and making positive statements about ourselves. If you are struggling with where to start, I encourage you to read the Casson lectures for inspiration. Find out what fits well with you and use that as your starting point for influencing change within the profession.

**Table 1.** Examples of the treasure trove of inspiration embedded in the Elizabeth Casson Lectures.

Casson Memorial Lecturer*	Focus	Ideas for how we can meet our hopes and aspirations as a profession
Godber (1973: 397)	• Elizabeth Casson: Her life and inspiration • Historical perspective	‘The case for occupational therapy has been made’.
Glanville (1975: 127)	• Elizabeth Casson: Her life and inspiration • Historical perspective	‘I do recommend you should read Elizabeth Casson’s article [Casson E (1941) Forty cases treated at the Allandale Curative Workshop, The Lancet], if you are interested in rehabilitation’.
Aitken (1976: 173)	• Historical perspective	‘Become involved in measurement’.
Mendez (1978: 228)	• Historical perspective; growth of the profession	‘We must put all our energies into ensuring that the added opportunities available to us are being fully utilised’.
Wright (1979: 166)	• Theoretical development:	‘What we say to patients has just as profound an effect as what we do for them or give to them’.
Atkinson (1980: 211)	• Elizabeth Casson: Her life and inspiration • Theoretical development	‘What is our *unique* contribution?’.
Fanshawe (1981: 194)	• Elizabeth Casson: Her life and inspiration • Theoretical development	‘. . .the occupational therapist is the person in the care team who is most likely to be able to provide a disabled person with the wherewithal for a satisfactory life. . .we seem to be sufficiently imaginative by nature to be able to turn a problem upside down and rethink the thing through’.
Farell (1982: 225)	• Theoretical development	‘. . .the profession needs to concentrate on establishing the extent of the effectiveness of existing programmes and the basis to that effectiveness; to determine the activities and techniques which are the most effective for the conditions and people to be treated’.
Bicknell (1983: 157)	• Theoretical development	‘. . .acknowledge not only the needs of disabled people but their rights and responsibilities as well’.
McLellan (1985: 232)	• Theoretical development	‘I very much doubt if she [Elizabeth Casson] would be satisfied. She does not seem to have been the sort of person to rest on her laurels. I feel sure she would have been making her presence felt in the establishment in a most uncomfortable and creative fashion today just as she did in 1930’.
Walker (1986: 146)	• Elizabeth Casson: Her life and inspiration • Historical perspective	‘. . .a greater opportunity for development presents now than ever before. If seized, I believe it could result in significant improvements in the quality of care we provide for patients, and in the job satisfaction of those providing it’.
Ellis (1987: 199)	• Elizabeth Casson: Her life and inspiration • Theoretical development	'Research is essential to maximise efficiency’.
Grove (1988: 152)	• Elizabeth Casson: Her life and inspiration • Historical perspective	‘Should the profession be fearful? Why should it? If occupational therapists are genuinely concerned to give as good a service as possible to all people who need it – within the resources that are likely to be available – then we must look more closely at what has to be done. Looking at it from a patient/client perspective, I want a prompt and effective service when I need it, where I need it’.
Fraser-Holland (1990: 327)	• Elizabeth Casson: Her life and inspiration • Historical perspective	‘convert our ideas and professional reasoning into written form’.
Barnitt (1991: 110)	• Call to action • Historical perspective	‘I cannot predict the future; I can only see the current issues and guess at the outcome. We are, however, not helpless; we can tackle the issues and influence the outcome. The world continues to offer glittering prizes to those who have stout hearts and sharp swords (Smith 1923)’.
Stewart (1992: 279)	• Peg Fulton: Her life and inspiration • Historical perspective	‘to move forward we need a sense of vision, a confidence in our professional base and in how we can each contribute to society’.
Correia (1993: 254)	• Historical perspective	‘A sound set of values should be developed which acknowledges real outcomes rather than superficial, numerical targets. Good supervision will need to be in place to ensure the efficient use of core skills and maintenance of standards of care. Good support systems will need to be in place to prevent staff from becoming isolated, stressed and vulnerable’.
Hagedorn (1995: 330)	• Theoretical development	‘We need to consolidate our scientific knowledge concerning people, occupations, environments, therapy and the therapist. We need clear explanations of what occupational therapy seeks to achieve, and how it does this. We need a model of the occupational nature of human beings. We need to understand how theory relates to practice. The responsibility for doing this does not rest solely with academics. It is the responsibility of each and every therapist to contribute to the development of professional knowledge as well as techniques and skills of practice’.
Yates (1996: 356)	• Increasing diversity	‘It is essential that both the College, the corporate body, and individual members take responsibility for the equality practices within the profession, and within the organisations for which they work’.
Eakin (1997: 294)	• Research	‘The challenge for the profession is to shift the balance, now and in the future, towards the integration of research and practice into a seamless whole for the benefit of those who use our services and for the development of our profession’.
Richards (1998: 300)	• Historical perspective	‘The profession needs to have a bigger view of the world; to look at what the nation needs of it. By responding positively and keeping a firm eye on patients' and clients' needs, cost-effectiveness and outcomes, the value of occupational therapy will be appreciated: not because you have set out to promote your role but because you are seen to produce the results’.
Tyldesley (1999: 361)	• Alice Constance Owens: Her life and inspiration • Historical perspective	‘Do not wait to see what you can get out of the Association, but with all your heart put wait you can into it . . . Let us be a proud and happy band of fellow workers, unselfishly working together for the common good. It is only by full cooperation of all for all that we can attain our object, the firm establishment and the future expansion of our great profession (Ross 1938, p. 6)’.
Mayers (2000: 365)	• Historical perspective • Future focus	‘respect the past and to embrace positively the future of our profession, occupational therapy’.
Roberts (2001: 497)	• Specialist practice • Future focus	‘We can develop the diversity of skill through cultivating an attitude of lifelong learning. Lifelong learners take risks. It is a matter of pushing ourselves out of the comfort zones and trying new ideas. The challenge is not for us to become set in our ways, but to keep experimenting’.
Willson (2002: 314)	• Research • Future focus	‘we have to look outwards and not only perceive the bigger picture but also take a part in shaping it’.
Walker (2003: 343)	• Research • Alice Constance Owens: Her life and inspiration • Historical perspective • Future focus	‘We must be open to new and exciting technologies, all of which need to be researched to establish if they are efficient and effective ways to treat our patients’.
Butler (2004: 289)	• Elizabeth Casson: Her life and inspiration • Theoretical development	‘We must have time to be and time to think’
Mountain (2005: 299)	• Elizabeth Casson: Her life and inspiration • Future focus	‘In a few decades’ time (depending upon your age now), if you are asked to recall what the main challenges are that you have faced during your career as an occupational therapist, what do you imagine that you will say? Will you be able to answer without hesitation? Will you be able to describe your achievements clearly? Will you be confident that you have made a difference?’.
Smith (2006: 307)	• Use of narratives	‘People’s narratives and stories are at the heart of change’.
Taylor (2007: 282)	• Increasing diversity	‘Let us celebrate and embrace diversity as an asset’.
Warren (2008: 275)	• Specialist practice • Research	‘Occupational therapists should be able to make a unique contribution because they are used to thinking about developmental progressions and personal goals, rather than about medical targets and cellular organisation, and their education prepares them to understand both physical and psychological needs in a unique way’.
Lawson-Porter (2009: 291)	• Future focus	‘. . .be radical and audacious’.
Drummond (2010: 298)	• Future focus • Research	‘You must decide how you wish to shape the profession. The way in which we choose to move forward is your decision, but it should be directed by research and by a focus on patient and client care’.
Turner (2011: 322)	• Theoretical development • Future focus	‘But if we return to work on Monday and begin to ask where we want our practice to go, how we are going to ensure it gets there and which factors support and inhibit occupation-based practice, we will have made a start’.
Ballinger (2012: 356)	• Rosemary Barnitt: Her life and inspiration • Research • Future focus	‘We need to become more politically astute in describing our skills and domains of practice to secure the future for our profession and for those who can benefit from our services’.
Hunter (2013: 353)	• Future focus • Historical perspective • Use of narratives	‘what would you do tomorrow if you knew you could not fail?’.
Hammond (2014: 392)	• Historical perspective • Specialist practice • Future focus	‘how therapists might need to change to implement evidence-based practice’.
Jackson (2015: 559)	• Use of narratives • Future focus	‘We must also have the courage to push boundaries and use our skills to take risks with people to enable them to make choices about their health and social care’.
Bryant (2016: 528)	• Elizabeth Casson: Her life and inspiration • Theoretical development • Research	‘It [occupational alienation] can enable reflection on our own sense of alienation and recognise who is alienated, whether occupationally, socially or existentially’.
Cox (2017: 531)	• Theoretical development • Research	‘As occupational therapy researchers and professionals in your field, consider writing that one piece that expands the evidence for occupational therapy each year, and find a place to publish – whether you are a researcher aiming at a journal, or a practitioner writing about your innovations in practice for a professional magazine, blog or newsletter’.
Pollard (2018: 493)	• Social transformation • Use of narratives	‘We should use our superdiverse status as a global city to build our strengths and express our breadth as a profession. Occupational therapy is familiar with narrative as a tool, but this can be expanded into a tool for inspiring, informing and involving people in actions at a personal and community level, kept alive by an inclusive cultural exchange’.
Kantartzis (2019: 563)	• Social transformation • Theoretical development	‘to ‘shift our focus’, look with courage at those problems that we believe we cannot change or that we do not know how to work with. We must join the conversations, embrace the power of occupation to be part of the processes of change, and work towards the transformation of our society towards health for all’.
Preston (2022: 10)	• Theoretical development • Research • Future focus	‘We need to move beyond a model of client-centredness in which we position the person at the centre of decision making to a genuine model of co-production in which we make better use of each other’s assets, resources and contributions to achieve better outcomes or improved efficiency (Bovaird and Löeffler, 2012)’.
Chu (2022: 629)	• Research • Historical perspective	‘it is important to measure performance outcomes (related to service efficiency), clinical outcomes (related to service effectiveness) and economic outcomes (related to cost-effectiveness)’.

*Note.* *No lecture was published in 1974, 1977, 1984, 1989, 1994, 2021, and 2023.

### We can all exert social leadership

Others describe feeling like they are not leaders. In the Social Age, lack of experience or skills does not matter. Whatever stage of our careers we are at, or what position we hold, we can engage in social leadership. Telling our stories relies upon authenticity, co-creation and networks of relationships, not position and hierarchy (Tempest and Dancza, 2019).

These new opportunities – of the Social Age – provide us with the powerful means to amplify our profession’s societal footprint. And the rather amazing part is that these new tools are both democratized and personalized – anyone can become an occupational therapy advocate and reach out to a potentially global audience (Jacobs, 2012: 654).

In terms of experience, people also cite being too experienced as a barrier, saying things like ‘You can’t teach an old dog new tricks’. Just as you cannot be too inexperienced, you cannot be too experienced either, as Professor Annie Turner’s (2021: para 3) reflection on writing her Casson lecture demonstrates

‘I think by the end I could finally say that I’d ‘got it’, that I truly understood what occupational therapy was about, that I could debate and defend its kaleidoscope of practice scenarios. But I had by then been practising for more than four decades and my path to this understanding had been convoluted and complex’.

Fundamentally, ‘We have to start recognising ourselves as leaders’ (Orman, 2018: para 7). For those who are put off because they feel they have tried in the past and been unsuccessful and so it’s not worth trying again, Darren McGarvey reminds us ‘It is important to recognise that you will fail, and fail many times. This is part of what it takes to change the world. We need to accept this and accept the consequences of failure, we will not always get it right’ (Bannigan, 2023: para 3). We need to persevere.

I fully appreciate it is easy for me to throw down a gauntlet and challenge others, especially from this platform, but as I mentioned earlier what I ask of you, I ask of myself so I also want to share what I am doing to increase the recognition of the centrality of occupation in my own work. The last section of the lecture will focus on EmpowerAge.

## The centrality of occupation to primary prevention

My story starts with another eminent occupational therapist, Ann Wilcock (2008), who, ahead of her time, understood occupational therapists, with our expertise in everyday activities and their impact on our habits, routines and roles, are well-placed to promote health. Whilst others agreed with Wilcock that we can ‘use our occupational perspective to deliver on population health and well-being’ (Lawson-Porter, 2009: 290), at the time of her death, Hocking (2020: 3) observed:

For herself, Ann was extremely modest about, even disappointed with, the impact her work is having on occupational science, occupational therapy theory and practice, population health, and on everyday people’s knowledge of occupation as the key to flourishing as individuals and societies

In Wilcock’s (2008: 34) own words ‘For some reason the profession failed to build upon the health-giving properties of doing wisely to enhance well-being, maintain health and prevent disease for the population at large’. Today we better understand that occupational therapists have a role to play in health promotion and public health (Allied Health Professions Federation, 2019). That said, two reviews of health promotion and public health in the Allied Health Professions have shown that occupational therapists have generally focused on secondary prevention (Fowler-Davis et al., 2020; Needle et al., 2011). For example, falls prevention for older adults following a fall. Secondary prevention, which focusses on intervening in the early stages of disease, where disease processes have begun, but not yet progressed in severity to cause symptoms (Fullerton et al., 2023: 67), is important. As is tertiary prevention, which involves managing a disease in the symptomatic phase, with the aim of limiting the impact, minimising disability and maximising quality of life (Fullerton et al., 2023: 67). For example, using an occupation-based intervention, like ‘Journeying through Dementia’, to support people with Dementia to engage in meaningful activities and maintain community connectedness (Craig et al, 2023: 207). However, a lack of focus on primary prevention is problematic. This is because the proportion of older people in society globally is predicted to double to 22% between 2015 and 2050 (World Health Organization, 2022), which means active aging and healthy lifestyles are priorities for public health (Rodríguez-Monforte et al., 2020).

### Healthy ageing

Healthy ageing is ‘the process of developing and maintaining the functional ability that enables well-being in older age’ (World Health organisation, 2015a). We know ageing well depends on lifestyle factors such as healthy eating, hydration, exercise, social connections and cognitive health (Vseteckova, 2023), which taken at face value does not immediately suggest a role for occupational therapy. However, achieving all these lifestyle factors depends on us making health-promoting decisions in relation to what we do every day. This suggests that focusing on what we do each day – doing wisely – is a good starting point for exploring lifestyle changes to support healthy ageing (Moll et al., 2015) indicating that occupational therapists have an important role to play in promoting healthy ageing (Knightbridge et al., 2022).

## EmpowerAge: An intervention for The Social Age?

Understanding this led me to developing a new health promotion intervention called EmpowerAge. It uses an occupation-based approach to healthy ageing for people living in the community, centred on what people do every day (Moll et al., 2015). Unsurprisingly, the ‘Do Live Well’ framework, which links doing – what people do every day – with health and well-being provides the conceptual framework for our intervention (Moll et al., 2015). Working with students on a research placement, during COVID-19, we decided to develop an intervention manual. The reason for this was that any students who would eventually deliver the intervention as part of their practice-based learning would fully understand what is involved. This is because a manualised intervention provides a detailed account of its components. Manualised interventions cover everything – the marketing, setting, recruitment, referral, assessment, outcome measurement, goal setting, intervention content and evaluation – as well as explaining all the underpinning theory. This is a pre-requisite first step in developing a new evidence-based intervention. An added advantage of manualisation is it makes an intervention more amenable to conducting successful evaluation studies. If there is a detailed description of what is involved in an intervention, it is easier to assess its fidelity (Blanche et al., 2011). The Medical Research Council has guidelines for developing and evaluating a complex intervention, the process captured in Skivington et al.’s (2021) summary of the new Medical Research Council’s guidelines. We used these guidelines to shape this work, and the first step is to identify if an intervention already exists or if we needed to develop a new one (Skivington et al., 2021).

When we started, we focused on the transition from work to retirement. Our theory was that life transitions offer an optimal moment to focus on lifestyle choices and well-being. This is because transitions have an influence on what people do and how they organise their daily activities (Jonsson, 2014). To this end, we conducted a rapid review of the literature (Bannigan et al., 2022). Of the 677 articles identified, 36 were included. The need for pre-retirement planning was widely discussed. However, no pre-existing, generic occupation-based intervention was identified. We did identify one intervention to support professional driver retirement transition for older taxi drivers in Singapore and, whilst it was not generalisable, it was encouraging to know that this type of intervention could be developed (Chan et al., 2015). The findings from studies and tools identified, such as tactical activity planning (Cantor, 1991), were used to develop a first draft of an intervention manual. Working with another group of students, when the COVID regulations allowed, we shared the manual with a Patient and Public Involvement and Engagement group – a PPIE group – in discussion with these groups we soon realised that, by focussing on the transition from work to retirement, we had distorted the focus of the intervention. There was a sense that the ‘Work to Retirement’ intervention was about ‘work identity’ rather than ‘healthy ageing’. This prompted us to take a step back and to ensure that the intervention was focussed on healthy ageing, after all people can live decades after they retire and healthy ageing will be one of the determinants of how well they live, including their ability to stay living in their own home (Mack et al., 1997). This enabled us to more fully emphasise the life course approach, which recognises that health and well-being are influenced by social, economic, and environmental factors throughout a person’s life, as well as personal characteristics (World Health organisation, 2015b).

### Occupational therapy and healthy ageing

With this shift in approach, and given that time had elapsed since our last review, we needed not only to revisit the literature but take a broader perspective on occupation and healthy ageing. A review by Jessen-Winge et al. (2018) identified a relationship between occupation and well-being for older people, based on evidence from three studies of moderate quality. The relationship seems to be related to a person having the opportunity to experience variation and independence in the activities they are involved in, having choice in the occupations they engage in and having a balance of activities that are done alone and with others (Jessen-Winge et al., 2018). Interestingly, despite the life course approach endorsed by the World Health Organisation (2021), the occupational therapy literature related to ageing focuses on older people between the ages of 60 (Stav et al., 2012) to 80 or older (Portillo et al., 2023). They also focussed on secondary prevention, within the context of disease management, rather than primary prevention with community-dwelling adults (Bannigan et al., 2024) confirming the reviews of public health and health promotion in the allied health professions discussed previously. In our search for existing reviews, we found 11 recent reviews but seven were on discrete topics, such as heatwaves (Fransson et al., 2023), joy (Goodwin et al., 2023) or e-tools for transport planning (Tahir et al., 2023), and no review identified the intervention characteristics of the included studies (Bannigan et al., 2024).

Understanding intervention characteristics is essential for developing an intervention (Gustafsson et al., 2009). This meant our next step was to conduct a scoping review (Bannigan et al., 2024), We completed it in January and are in the process of writing it up for publication. Our findings will be used to revise our manual and the manual will be drafted in line with the Template for Intervention Description and Replication – known as the TIDieR guidelines (Hoffmann et al., 2014). Using the TIDieR guidelines is best practice and supports replication. An RCOT Innovation Award has been secured to support the write-up and the work will be completed in May. Then in June, the manual’s acceptability will be evaluated, by another group of students on a research placement, using cognitive interviewing with the different stakeholder groups. Our aim is for EmpowerAge to be offered as a health promotion placement within a practice-based learning module in the next academic year. This will give us the opportunity to further test its acceptability and start planning for feasibility testing. Whilst this is a work in progress, we envisage that EmpowerAge will be a core intervention that can be used to promote healthy ageing across the life course. It will be tailored to different age groups and people in different sectors in health and social care and beyond. This means life transitions (such as moving from primary to high school or work to retirement) may provide optimal moments for offering the intervention, but equally it could be tailored to target specific populations such as carers or people in prison without losing the core focus on healthy ageing.

### EmpowerAge: Intervention development in the social age

From a Social Age perspective, EmpowerAge has been a wonderful opportunity to share how occupation is central to the lives of people and communities. After all, we all know that exercise, hydration and diet are important to health but how many of us do it, and do it consistently? This is where our expertise as occupational therapists comes to the fore. By starting with the person, their life, and how they spend their time and then working together to explore how they can make healthier choices, they can start to make choices in a way that makes sense to the way they live their lives and the challenges they face. Discussing primary prevention has been a fantastic opportunity for me to discuss the contribution of occupation to people’s lives. By manualising EmpowerAge and describing explicitly the mechanisms of change, we are delineating how occupational therapy achieves change. By offering EmpowerAge as a health promotion placement to students, we also are ensuring that before they graduate students are conversant with primary prevention and the role of occupation in achieving it.

Working on EmpowerAge has also demonstrated the importance of developing relationships with, and working with, the communities who we serve. The early involvement of people who have recently retired and a PPIE group in our work highlighted a flaw early on. This flaw may have derailed the project at the implementation stage if we had not chosen to work with the people the intervention is targeted at. It has also been important to recognise the range of stakeholders in the project – the local population, students and occupational therapists – who all have different interests in, and a perspective on, the project. We are building relationships within each community. The logo, which was designed with our PPIE group and students, has given the project a strong visual identity which makes our story easier to share. For example, the logo was recently used as the article thumbnail picture on one of our recent publications. It will also support social media activity related to EmpowerAge.

## Conclusion

This discussion of EmpowerAge demonstrates that what is required of us is different to what was asked of Elizabeth Casson. We live in a different age and experience different challenges. Re-reading Reilly’s (1962) Eleanor Clarke Slagle lecture heartened me; occupational therapy is in great shape. We have come a long way but, given the centrality of occupation in people’s lives, there is so much more we could be achieving. We have a great story to share and a social movement, built on a profession that is the size of a global city (Pollard, 2018), means our potential for impact is greater than it has ever been. The Elizabeth Cassons who live amongst us today are the living embodiment of our story. Each and every one of us needs to find our ‘inner’ Elizabeth Casson whether it is adding new plot lines to our story or telling our story. Only you can make the choice about what you will do, only you can decide to engage, no one else can make you play your part. What are you going to do to release your inner Elizabeth Casson? ‘It does take courage, heart, brains and spirit to survive in a professional world that is constantly challenging and rewarding’ (Roberts, 2001: 193). I wish that courage for us all. We need to place the worries of the past in the past and stop being absorbed with how we are perceived as a profession. Once others understand the centrality of occupation, we can start directing our energies towards the people and communities we serve. To steal the closing words of another Casson lecturer, ‘If we can agree that collective action is possible and desirable then can I suggest that there is no time like the present, there is nothing to stop us except ourselves – so why don’t we just get on with it?’ (Lawson-Porter, 2009: 292). The time is now.

I am deeply honoured to have had the privilege of this opportunity. Thank you for your interest.

**Key findings**

- The social age has democratised the processes for achieving reach and impact.
- Social movements, a form of collective action, provide a means for increasing the centrality of occupation within society.

**What the study has added**

Social movements and social leadership rely upon individuals choosing to act and, through this collective action, increase the reach of ideas, such as the centrality of occupation in people’s lives.
